# Potential mechanisms underlying the therapeutic roles of sinisan formula in depression: Based on network pharmacology and molecular docking study

**DOI:** 10.3389/fpsyt.2022.1063489

**Published:** 2022-11-09

**Authors:** Hui Wang, Jiaqin Liu, Jinbiao He, Dengxia Huang, Yujiang Xi, Ting Xiao, Qian Ouyang, Shiwei Zhang, Siyan Wan, Xudong Chen

**Affiliations:** ^1^Department of Psychiatry, National Clinical Research Center for Mental Disorders, The Second Xiangya Hospital of Central South University, Changsha, China; ^2^Yunnan University of Traditional Chinese Medicine, Kunming, China; ^3^Department of Pharmacy, The Second Xiangya Hospital, Central South University, Changsha, China; ^4^Institute of Clinical Pharmacy, Central South University, Changsha, China; ^5^The First Affiliated Hospital of Hunan University of Chinese Medicine, Changsha, China; ^6^Hunan University of Chinese Medicine, Changsha, China

**Keywords:** sinisan formula, depression, network pharmacology, molecular docking, neurotransmitter-related mechanisms

## Abstract

**Background:**

The incidence of depression has been increasing globally, which has brought a serious burden to society. Sinisan Formula (SNSF), a well-known formula of traditional Chinese medicine (TCM), has been found to demonstrate an antidepressant effect. However, the therapeutic mechanism of this formula remains unclear. Thus, the present study aimed to explore the mechanism of SNSF in depression through network pharmacology combined with molecular docking methods.

**Materials and methods:**

Bioactive compounds, potential targets of SNSF, and related genes of depression were obtained from public databases. Essential ingredients, potential targets, and signaling pathways were identified using bioinformatics analysis, including protein-protein interaction (PPI), the Gene Ontology (GO), and the Kyoto Encyclopedia of Genes and Genomes (KEGG). Subsequently, Autodock software was further performed for conducting molecular docking to verify the binding ability of active ingredients to targets.

**Results:**

A total of 91 active compounds were successfully identified in SNSF with the use of the comprehensive network pharmacology approach, and they were found to be closely connected to 112 depression-related targets, among which CREB1, NOS3, CASP3, TP53, ESR1, and SLC6A4 might be the main potential targets for the treatment of depression. GO analysis revealed 801 biological processes, 123 molecular functions, and 67 cellular components. KEGG pathway enrichment analysis indicated that neuroactive ligand-receptor interaction, serotonergic synapse pathways, dopaminergic synapse pathways, and GABAergic synapse pathways might have played a role in treating depression. Molecular docking suggested that beta-sitosterol, nobiletin, and 7-methoxy-2-methyl isoflavone bound well to the main potential targets.

**Conclusion:**

This study comprehensively illuminated the active ingredients, potential targets, primary pharmacological effects, and relevant mechanism of the SNSF in the treatment of depression. SNSF might exert its antidepressant effects by regulating the signaling pathway of 5-hydroxytryptamine, dopamine, GABA, and neuroactive ligand receptor interactions. Still, more pharmacological experiments are needed for verification.

## Introduction

Depression has been recognized as a major global public health issue due to its significant morbidity and mortality, cognitive and behavioral changes, psychotic symptoms, and even self-injury and suicidal behaviors. According to the World Health Organization (WHO), major depressive disorder (MDD) is the third leading cause of global burden and is expected to be the first leading cause by 2030 (1). Evidence indicates that depression is often associated with major economic crises and natural disasters (2, 3); for example, the COVID-19 pandemic has had a great impact on the mental health of people in many regions (4, 5). Statistics have shown that there were approximately 53.2 million new cases of major depressive disorder worldwide in 2020 due to the COVID-19 pandemic (6), and patients who have had COVID-19 were at a higher risk of brain fog and depression even 1 year after infection (7).

To date, the pharmacological treatment of depression mainly includes selective serotonin reuptake inhibitors (SSRIs), serotonin and norepinephrine reuptake inhibitors (SNRIs), and monoamine oxidase inhibitors (MAOIs), which have been demonstrated to be effective on depressive symptoms. However, one-third to half of the patients with depression was found to be unresponsive to antidepressant agents (8). Many patients reported adverse effects such as gastrointestinal reactions, hepatotoxicity, weight gain, metabolic disturbances, sexual dysfunction, which are some of the most common factors leading to non-compliance and discontinuation of treatment (9, 10). According to a study, about 43% of patients with MDD discontinued their antidepressants due to emergency adverse reactions (11). At times, the adverse reactions do not necessarily subside after the discontinuation of antidepressants, which might lead to altered responsiveness to subsequent treatments (12). Safety and suicidality are also major concerns. Risk factors such as overdose, pregnancy, and breastfeeding may also limit the use of antidepressants. Therefore, alternative therapies with better efficacy and safety are urgently needed to avoid the long-term use of antidepressants and to reduce adverse effects.

Recently, herbal remedies and plant products have been increasingly used to treat depression (13). A growing body of evidence has demonstrated that many phytochemicals, such as flavonoids, saponins, alkaloids, polyphenols, triterpenoids, and fatty acids, produce anxiolytic and antidepressant-like effects (14). Traditional Chinese medicine (TCM), a commonly used complementary and alternative therapy, has demonstrated significant therapeutic effects among patients with MDD (15). Sinisan Formula (SNSF), a classical TCM formula first described in “Shang Han Lun (AD198–201),” consists of four botanical drugs: *Bupleurum chinense* (Chaihu), *Paeoniae Radix Alba* (Shaoyao), *Aurantii Fructus Immaturus* (Zhishi), and *licorice* (Gancao), with a dose proportion of 1:1:1:1. According to the theory of TCM, SNSF has been widely used in treating liver stagnation and spleen deficiency, disorders of the digestive system, as well as depression for thousands of years in China.

Previous studies have shown that SNSF has the potential to treat cardiovascular diseases, nutritional and metabolic disorders, and psychiatric disorders. With a variety of active ingredients, SNSF has been increasingly used in clinical practice due to its significant effects on MDD. Chaihu and Shaoyao, two essential herbs in SNSF, are one of the most classic compatible TCM drug pairs in soothing emotions and have a significant regulatory effect on the monoaminergic neurotransmitter system, suggesting their antidepressant effect (16). A study showed that SNSF exerts an antidepressant effect by improving synaptic plasticity *via* the CaSR-PKC-ERK signal pathway in rats exposed to stress (17); this formula also exhibited therapeutic effects on animal models with depression-like behavior induced by early life stress (18). Previous studies in network pharmacology have predicted the molecular mechanism of SNSF in treating neurological and mental disorders, which mainly included regulation of neuronal activity through neurotransmitters, nervous protection, and influence on neuronal migration and differentiation (19). Although SNSF has a long history of use, its anti-depressive active ingredients, putative target genes, and underlying mechanisms have not been fully clarified.

In recent years, network pharmacology has been proposed as a promising approach in studies of TCM; it is based on the biomolecule network of drug targets and disease-related targets to establish the correlation between drugs and diseases at the system level and the biological network level as a whole (20). Molecular docking is a method for the identification of molecular interactions and the prediction of the structure of receptor-ligand complexes by simulating molecular geometry and intermolecular forces between receptors and ligands, which improves the efficiency of chemical activity evaluation and the orientation of compound discovery as well as provides a new method for further mining of the TCM resources (21).

Herein, with the help of network pharmacology and molecular docking technology, the present study aimed to explore (1) the active ingredients involved in the treatment of depression, (2) the protein regulatory goals to achieve the biological activity, (3) the biological processes or pathways by which the active ingredients treat depression, and (4) the molecular docking binding ability and key targets of the active ingredients by investigating the therapeutic effect of SNSF on MDD, in order to provide a basis for relevant connotation interpretation, theoretical studies and clinical precision application of this promising herbal formula.

## Materials and methods

### Acquisition of bioactive compounds of sinisan formula

The information on all ingredients of SNSF was acquired from the Traditional Chinese Medicine Systems Pharmacology Database and Analysis Platform (TCMSP^1^, updated version, June 2nd, 2021), which is specifically designed to characterize the relationships between drugs, targets and diseases of Chinese herbal medicines (22). According to the literature, pharmacokinetic parameters such as absorption, distribution, metabolism, and excretion (ADME) are essential contributors to the biological activity of a drug. Compounds with oral bioavailability (OB) ≥ 30% were considered to have good absorption and slow metabolism after oral administration, while compounds with drug-likeness (DL) ≥ 0.18 were considered to be important indicators for the evaluation of ADME properties; thus, the two parameters were used as crucial factors to screen out pharmaceutically active compounds (23). Meanwhile, as the blood-brain barrier (BBB) is the most selective and tight barrier throughout the body, the effect of a drug to regulate cognitive or mental disorders might be affected by its ability to penetrate the BBB, i.e., with a permeability of more than −0.3 (24). Thus, OB, DL, and BBB were selected parameters to identify the potentially active compounds in SNSF.

### Acquisition of herb-disease genes

All target proteins were converted to their corresponding gene symbols in the UniProt database^2^ and the species “Homo sapiens” was used to normalize gene names. The keyword “depression” with “Homo sapiens” genes only was searched in databases of GeneCards^3^, Disgenet^4^, TTD^5^, OMIM (Online Mendelian Inheritance in Man^6^), and DrugBank.^7^ After the deletion of duplicate genes, active compound targets were mapped to depression-related targets, and therapeutic targets of SNSF against depression were obtained using a Venny2.1 online tool.

### Protein-protein interaction network

The targets shared by the herbs and disease were then entered into the Search Tool of the Retrieval of Interacting Genes database (String^8^) for PPI network construction. The topological properties of targets were analyzed using the CytoNCA tool (a plug-in of Cytoscape), in order to identify key target genes with high connectivity with SNSF. Three parameters, namely degree centrality (DC), betweenness centrality (BC), and closeness centrality (CC), were estimated using the Network Analyzer tool for topological analysis. DC is a key topological parameter representing the number of nodes in the relations between one node and other nodes in a network; The degree value is used to describe the significance of potential targets and active ingredients, with ingredients with higher degrees indicating that more targets would respond to it. Meanwhile, targets with a higher degree value also indicated that they might be the primary targets in the molecular mechanism.

### Protein-protein interaction enrichment analysis

The protein-protein interaction enrichment analysis was conducted using the STRING, BioGrid, OmniPath, and InWebIM databases, while the analysis of physical interactions was performed using only STRING (physical score > 0.132) and BioGrid. The network was obtained through the analysis involving the subset of proteins that had physical interactions with at least one other member in the list. For networks consisting of 3 to 500 proteins, the Molecular Complex Detection (MCODE) algorithm should be applied to identify the densely connected components in the network.

### Gene ontology functional annotation and Kyoto encyclopedia of genes and genomes pathway enrichment analysis

Gene ontology (GO) function annotation^9^ and KEGG pathway enrichment analysis^10^ were performed to examine the mechanism of SNSF in the treatment of depression and to clarify the biological processes involving target proteins in distinct clusters, as well as their involvement in signaling transduction. After entering the target genes and restricting the species to humans exclusively, all of the target genes were adjusted to their official gene symbols. The GO terms and KEGG pathways were regarded as significant when the *p*-value was lower than 0.01 after Bonferroni correction. We employed FDR multiple comparison adjustment to avoid false positives, as we conducted in our previous study (20).

### Compound-target molecular docking verification

Molecular docking is an essential technology for verifying compound-target interactions in network pharmacology and evaluating the binding affinity (25, 26). In the present study, the top ten candidate target proteins were selected as the core target for molecular docking analysis based on degree values in the PPI network. The molecular docking was performed using AutoDock Vina (version 1.5.6), which is extremely widely used in the field of pharmaceutical small molecule and biomolecule science and has been validated the superiority of speed, accuracy, and usability to provide unique positive enrichment rates for high-throughput virtual screening (27, 28). The 3D structures of proteins were retrieved and downloaded from the Protein Data Bank (PDB)^11^ and then saved as PDBQT protein receptor format files after the removal of water molecules, co-crystalline ligands, and ions by Pymol software. The 2D structures of compounds were retrieved and downloaded from the PubChem database,^12^ then transferred to 3D structures after energy minimization by Chem3D software. Vina software was utilized to conduct molecular docking and calculate docking affinity. The specific steps and parameters of docking were the same as those in one of our previous studies (29). Pymol (3D) and LigPlus (2D) softwares are employed to visualize the docking results, including the docking site as well as the patterns of hydrogen bonding interactions between the ligand and the main chain or side chain elements of the protein.

## Results

### Screening of active compounds of sinisan formula

**FIGURE 1:**
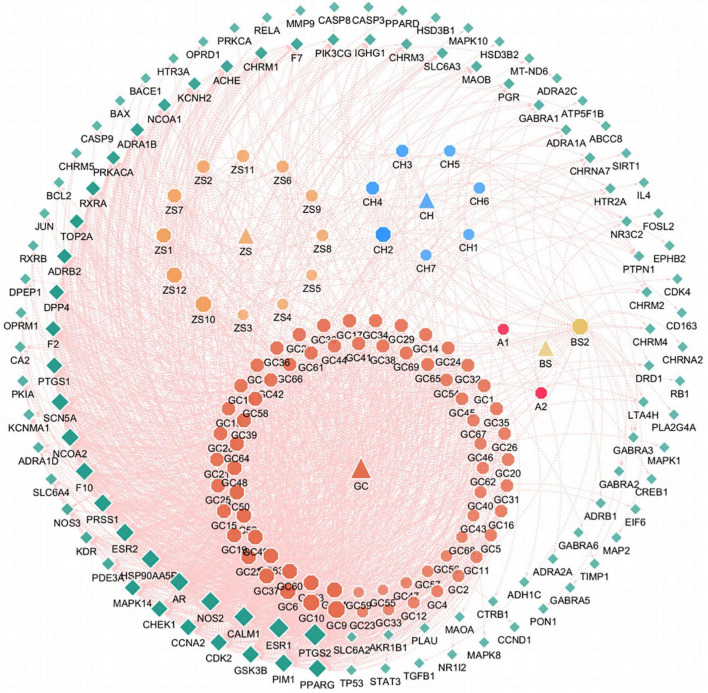
Compound-target network of sinisan formula (SNSF). The green squares represent the genes, and the orange, blue, gold octagonals represent the active compounds of SZHP. A1 and A2 represent the common compounds of Gancao and Baishao. The pink lines represent the interaction between compounds and targets. Larger shapes and darker colors indicate stronger interaction.

A total of 91 potentially active ingredients in SNSF and 112 targets were identified, indicating that one compound might regulate multiple targets (Supplementary Table 1), such as isosinensetin, formononetin, nobiletin, stigmasterol, beta-sitosterol, and sitosterol, suggesting the multi-ingredient, and multi-target feature of SNSF in the treatment of depression. This finding also indicated that a compound might exist in different herbs, while one herb could contain multiple active components; this “many-to-many” relationship might be an essential basis for the multi-target feature of TCM. The corresponding genes were then extracted in the Uniprot database by checking targets. The active compounds and target genes are presented in Figure 1.

### Core target acquisition

**FIGURE 2:**
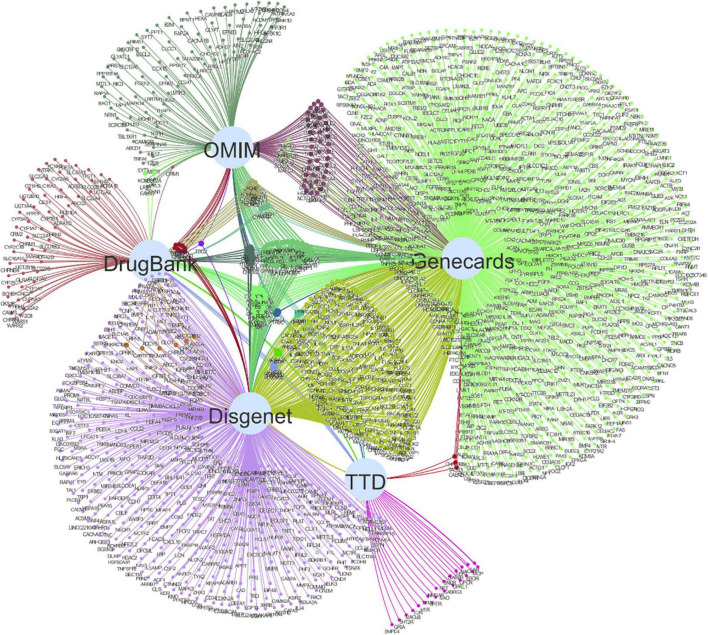
Related genes obtained from GeneCards, Disgenet, TTD, OMIM, and DrugBank databases.

**FIGURE 3:**
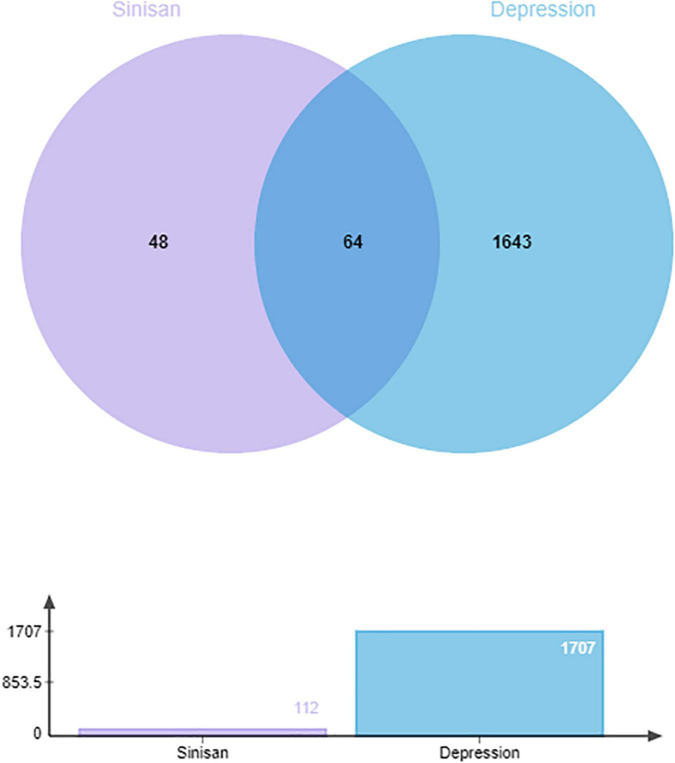
The Venn diagram involving the 1,707 targets screened out from databases, including 112 targets of sinisan formula (SNSF). The intersections indicate the 64 targets of SNSF for depression.

After searching and screening, 1,171 related genes were retrieved from the GeneCards database, 650 were retrieved from the Disgenet database, 40 were retrieved from the TTD database, 192 were retrieved from the OMIM gene database, and 123 were retrieved from the DrugBank database. After the deletion of duplicate genes, a total of 1,707 genes associated with depression were identified (Figure 2). A Venn diagram was plotted by inputting the identified drug targets and disease targets into the Venny software, and a total of 64 shared targets were obtained. See Figure 3 for details.

### Protein-protein interaction network construction and topological analysis of the drug-disease-target connection

**FIGURE 4:**
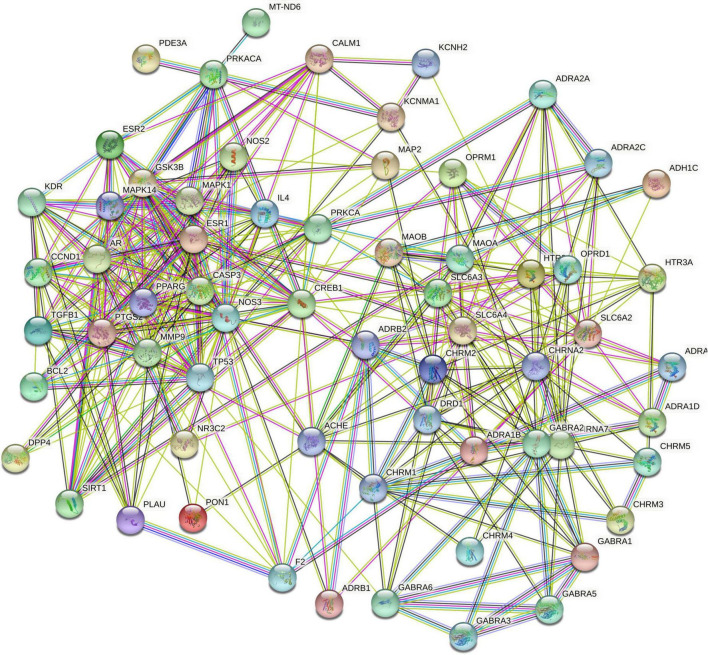
Protein-protein interaction (PPI) network of common targets of sinisan formula (SNSF) and major depressive disorder (MDD).

The targets shared by drug and disease were entered into the String database for PPI network construction (Figure 4), and the network was then imported into Cytoscape for topological analysis using Network Analyzer. The network consisted of 64 nodes and 374 edges, with a mean connection degree of 11.7. The DC was used as the reference standard, and the genes with scores higher than the mean score were selected as key targets. Finally, a total of 34 key targets were identified; the ten targets with the highest connectivity values are presented in Table 1.

**TABLE 1:** Top 10 key targets of protein-protein interaction (PPI).

Targets	DC	BC	CC
CREB1	28	0.127001815	0.617647059
NOS3	25	0.063178734	0.557522124
CASP3	24	0.043311073	0.547826087
TP53	23	0.044332505	0.533898305
ESR1	23	0.052436137	0.567567568
SLC6A4	22	0.086796242	0.583333333
MMP9	22	0.011538361	0.512195122
PTGS2	21	0.013835235	0.520661157
SLC6A3	19	0.062478141	0.567567568
GSK3B	19	0.01638299	0.525

### Gene ontology enrichment analysis

**FIGURE 5:**
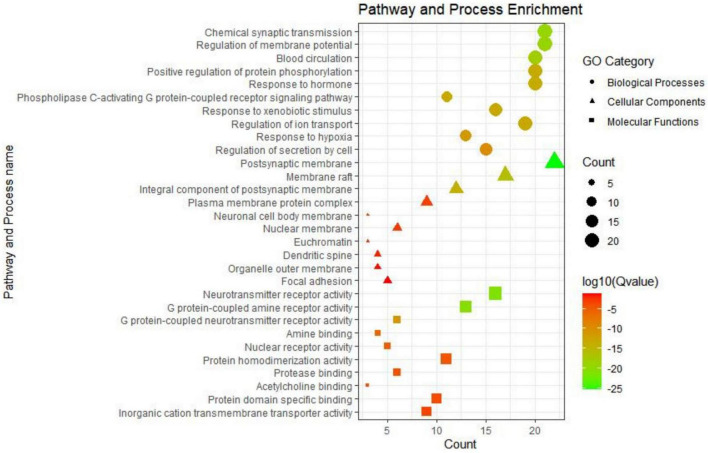
Biological processes (BP), cellular components (CC), and molecular functions (MF) of Go enrichment analysis.

A total of 801 biological processes (BP), 123 molecular functions (MF), and 67 cellular components (CC) were enriched. Most GO terms of BP were related to chemical synaptic transmission, regulation of membrane potential, blood circulation, positive regulation of protein phosphorylation, response to hormones, phospholipase C-activating G protein-coupled receptor signaling pathway, response to xenobiotic stimulus, regulation of ion transport, response to hypoxia, regulation of secretion by cell, etc. MF enrichment was mainly involved in the neurotransmitter receptor activity, G protein-coupled amine receptor activity, G protein-coupled neurotransmitter receptor, active amines, nuclear receptor binding, protein homodimerization, protease binding, acetylcholine binding, protein domain specific binding, inorganic cation transmembrane transporter activity, etc. The main terms of CC were associated with postsynaptic membrane, membrane raft, integral component of postsynaptic membrane, plasma membrane protein complex, neuronal cell body membrane, nuclear membrane, euchromatin dendritic spine, organelle outer membrane, focal adhesion, etc. The terms of BP, CC, and MF were ranked on the basis of their *Q* value, and the top 10 terms of each category are shown in Figure 5.

### Kyoto encyclopedia of genes and genomes pathway enrichment

**FIGURE 6:**
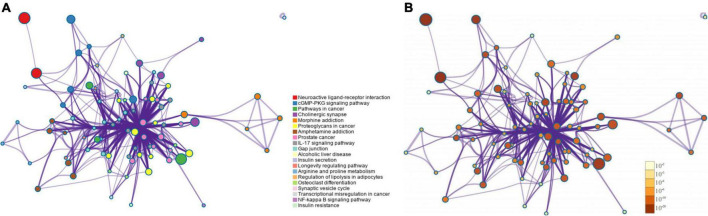
The top 20 enriched Kyoto encyclopedia of genes and genomes (KEGG) pathways: **(A)** colored by cluster ID, in which nodes sharing the same cluster ID are generally close to each other; **(B)** colored by *p*-value, in which terms containing more genes tended to have a more significant *p*-value.

**FIGURE 7:**
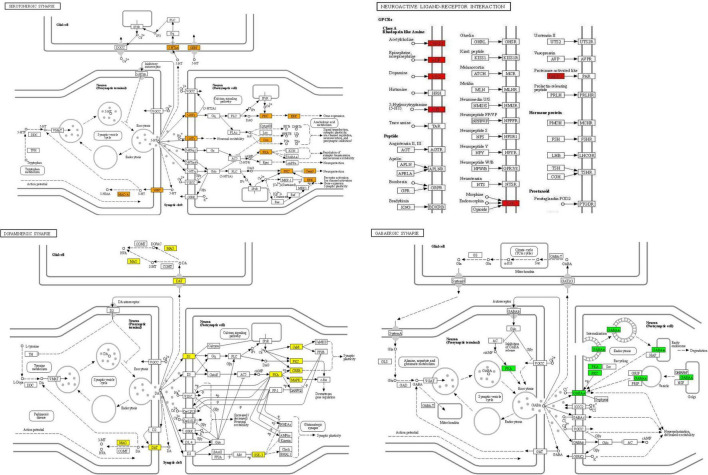
Nervous system interaction effects of the Kyoto encyclopedia of genes and genomes (KEGG) enrichment pathway terms and putative target proteins identified for SNSF. Red, orange, yellow, and green rectangles represent the putative target proteins identified in this study. Arrows indicate activation and round-arrows indicate inhibition.

In the KEGG pathway enrichment, a total of 153 signaling pathways were enriched after FDR correction, with the most significant pathways including the neuroactive ligand-receptor interaction, cGMP-PKG signaling pathway, pathways in cancer, cholinergic synapse, morphine dependence, etc. After hierarchical clustering of the enriched pathways, the networks with a similarity of > 0.3 were regarded as sub-networks, with the most significant pathway used as the reference of the enriched networks. Nervous system interaction effects of the KEGG enriched pathways and putative target proteins were identified for SNSF. Serotonergic synapse pathways (CASP3, HTR2A, HTR3A, MAOA, MAOB, PRKACA, PRKCA, MAPK1, PTGS2, and SLC6A4), dopaminergic synapse pathways (CALM1, CREB1, MAPK14, DRD1, GSK3B, MAOA, MAOB, PRKACA, PRKCA, and SLC6A3), and GABAergic synapse pathways (GABRA1, GABRA2, GABRA3, GABRA5, GABRA6, PRKACA, and PRKCA) were identified in this study. See Figures 6, 7 for details.

### Molecular functions of protein-protein interactions in enrichment

#### Molecular complex detection analysis

**FIGURE 8:**
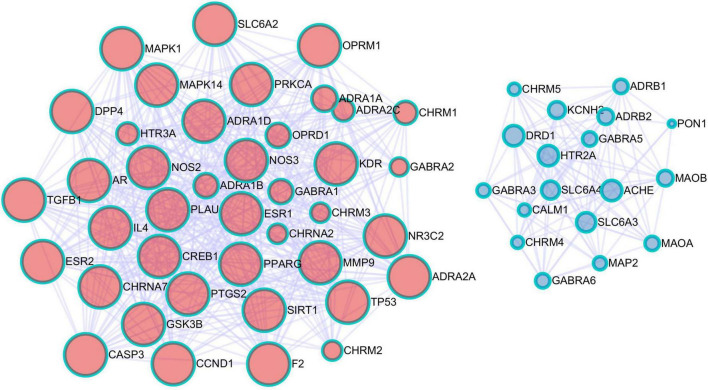
The top two gene clusters in the enrichment molecular complex detection (MCODE) analysis.

Key genes were identified through network construction and MCODE analysis using the complete datasets of STRING, OmniPath, InWebIM, and BioGrid for the independent enrichment analysis of gene clusters. The enrichment analysis of molecular functions was used for each MCODE component (Figure 8 and Table 2). The three terms with the highest scores based on the *p* values were used as the functional description of the corresponding components (Table 3).

**TABLE 2:** Molecular functions of protein-protein interaction in the enrichment.

MCODE	GO	Description	Log10 (P)
MCODE 1	GO:0008227	G protein-coupled amine receptor activity	−14.6
	GO:0004936	Alpha-adrenergic receptor activity	−13.7
	GO:0004935	Adrenergic receptor activity	−12.4
MCODE 2	GO:0030594	Neurotransmitter receptor activity	−15.0
	GO:0098960	Postsynaptic neurotransmitter receptor activity	−14.4
	GO:0008227	G protein-coupled amine receptor activity	−10.0

**TABLE 3:** The top three molecular functions in enrichment molecular complex detection (MCODE) analysis.

GO	Description	Log10 (P)
GO:0030594	Neurotransmitter receptor activity	−24.8
GO:0098960	Postsynaptic neurotransmitter receptor activity	−24.5
GO:0008227	G protein-coupled amine receptor activity	−23.7

### Molecular docking verification

**FIGURE 9:**
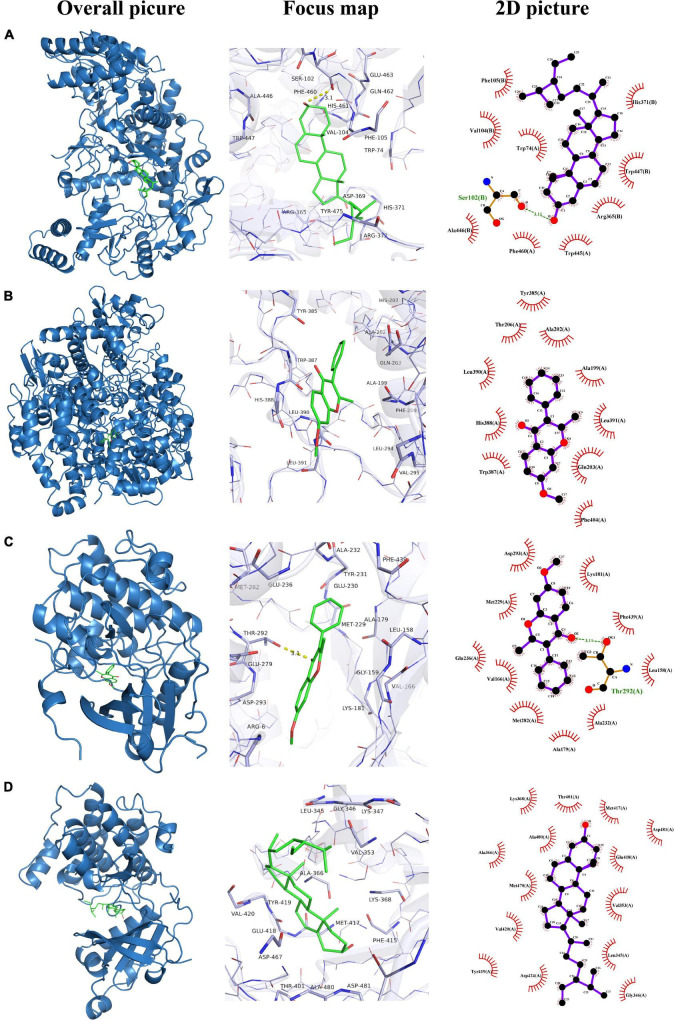
Molecular models of active compounds binding to the predicted targets. **(A)** NOS3 and beta-sitosterol; **(B)** PTGS2 and 7-methoxy-2-methyl isoflavone; **(C)** GSK3B and 7-methoxy-2-methyl isoflavone; **(D)** PRKCA and beta-sitosterol.

Ten candidate target proteins, including GSK3B(PDB ID: 1O6L), PTGS2(PDB ID: 3HS5), NOS3(PDB ID: 1M9M), PRKCA(PDB ID: 4RA4), SLC6A4(PDB ID: 5I6X), ESR1(PDB ID: 1L2I), MMP9(PDB ID: 2OVX), TP53(PDB ID: 2VUK), CASP3(PDB ID: 1NME), and CREB1(PDB ID: 5ZK1), were conducted molecular docking with three candidate bioactive compounds (7-methoxy-2-methyl isoflavone, beta-sitosterol, and nobiletin). The binding energy is generally considered to be less than −5.0 kcal/mol or −7.0 kcal/mol, indicating good or strong binding activity between the ligand and the receptor, respectively (30). The docking of GSK3B and 7-methoxy-2-methyl isoflavone had the lowest binding energy (–9.6 kcal/mol), the docking of CREB1 and nobiletin had the highest binding energy (–5.8 kcal/mol). In the present study, molecular docking results showed that the conformation of the active compounds and the main protein targets showed strong and reliable binding interactions. The docking results of binding affinity and detailed compound-target interactions are presented in Table 4 and Figure 9, respectively.

**TABLE 4:** The binding ability of active compounds to core targets.

Target	Active compound	PDB ID	Binding affinity with active compound (kcal/mol)	Binding affinity with original ligand (kcal/mol)
GSK3B	7-methoxy-2-methyl isoflavone	1O6L	−9.6	−9.5
PTGS2	7-methoxy-2-methyl isoflavone	3HS5	−9	−7.7
NOS3	beta-sitosterol	1M9M	−8.5	−7.1
PRKCA	beta-sitosterol	4RA4	−8.0	−9.6
SLC6A4	beta-sitosterol	5I6X	−7.6	−8.4
ESR1	nobiletin	1L2I	−7.1	−9.1
MMP9	nobiletin	2OVX	−7.0	−11.3
TP53	nobiletin	2VUK	−6.9	−6.1
CASP3	beta-sitosterol	1NME	−6.4	−5.7
CREB1	nobiletin	5ZK1	−5.8	/

## Discussion

Major depression disorder severely limits the social and psychological function and lowers the quality of life of patients, and it is often associated with a variety of chronic physical diseases, including hypertension, cardiovascular diseases, cancer, diabetes, obesity, cognitive impairment and dementia (31). With the increasing prevalence and morbidity, MDD has become a critical global concern of public health. SNSF is an established traditional medicinal formula and has displayed a variety of biological and pharmacological activities in the treatment of depression. To investigate the mechanism of SNSF in the treatment of MDD, this study was conducted using the network pharmacology approach and molecular docking.

In the active compounds-targets network, a total of 112 targets affected by 91 bioactive compounds in the SNSF were obtained. The core active compounds mainly included flavonoids such as isosinensetin, formononetin, nobiletin and sterols such as stigmasterol, beta-sitosterol, and sitosterol. Formononetin is a type of isoflavone that upregulates the expression of the glucocorticoid receptor and brain-derived neurotrophic factor, protects neurons, and promotes neurogenesis in the hippocampus, thereby effectively reducing chronic corticosterone-induced depression-like behavior in mice (32). Nobiletin not only upregulates synaptic neurotransmission, improves memory impairment and monoamine function, but also produces antidepressant-like effects through the interaction of the serotonergic, noradrenergic and dopaminergic systems (33). Some studies have shown that stigmasterol might effectively treat depression and anxiety disorders by reverting the imbalance of endogenous neuroactive steroids (34). According to an animal study, β-sitosterol was associated with a significant increase of NE, 5-HT, and the metabolite 5-HIAA in the mouse brain, suggesting that the antidepressant-like effect might have been mediated by these neurotransmitters (35). In summary, the main active ingredients of SNSF have different therapeutic effects on depression, involving nervous system interaction and multiple signaling pathways. However, our study also found that some other active ingredients have not been found in treating depression, which might be potential subjects to explore the treatment of depression in the future.

The PPI network analysis showed that the common targets included CREB1, NOS3, CASP3, TP53, ESR1, SLC6A4, MMP9, PTGS2, SLC6A3, and GSK3B. The cyclic adenosine monophosphate (cAMP) responsive element-binding protein 1 (CREB1), as a junction of intracellular depression-related signal transduction pathways, is key to the cAMP signaling pathway, while this pathway has been found to be altered in most patients with psychiatric disorders (36). Furthermore, CREB1 is an important mediator for the effects of antidepressants on the function and activity of the hippocampus (37, 38). In an experiment on mice, chronic stress stimulation led to decreased neurogenesis as well as neuronal cell damage and apoptosis along with the occurrence of depressive behaviors by increasing CASP3 activity and upregulating caspase activity (39). Estrogen is also closely associated with depression. Studies have shown that women, especially those in the menopause transition, are at a higher risk of depression (40–42). Estrogen receptor α (ESR1) is one of its main specific receptors involved in the mediation of the physiological function of estrogen, and it plays a vital role in regulating neurotransmitter conversion, including elevation of levels of serotonin and norepinephrine (43). Induced by lipopolysaccharides in the brain and proinflammatory cytokines, prostaglandin-endoperoxide synthase 2 (PTGS2) is mainly expressed in the hippocampus and glutamatergic neurons, where it helps maintain synaptic function, long-term synaptic plasticity and neurovascular coupling during functional hyperemia (44). Furthermore, with the gradual discovery of the link between depression and inflammation, the PTGS2 inhibitors have been demonstrated to facilitate the therapeutic effects of antidepressants (45). Taken together, our findings indicated the multi-target feature of SNSF in the treatment of depression and these targets might be the main potential targets for depression.

The GO function analysis showed that the therapeutic effect of SNSF in depression was mainly exhibited as chemical synaptic transmission, regulation of membrane potential, neurotransmitter receptor activity, G protein-coupled amine receptor activity, postsynaptic membrane raft, etc., while the MCODE analysis showed that neurotransmitter receptor activity, postsynaptic neurotransmitter receptor activity, and G protein-coupled amine receptor activity were the most correlated gene clusters. The KEGG pathway analysis showed that the targets of active components of SNSF were mainly concentrated in neuroactive ligand-receptor interaction, including serotonergic synapse pathways, dopaminergic synapse pathways, GABAergic synapse pathways, and cGMP-PKG signaling pathway, etc.

The monoamine neurotransmitter and receptor hypothesis of depression proposed lately suggests that the imbalance of monoaminergic neurotransmission is central to the biological dimension of depression, which mainly manifests as the reduction of the synthesis and release of neurotransmitters such as norepinephrine (NE), 5-hydroxytryptamine (5-HT), and dopamine (DA) in relevant brain regions (46). The serotoninergic synaptic pathway is composed of 5-HT and various serotonin receptors on the synaptic membranes in different regions of the brain, and most of these receptors belong to the G protein-coupled receptors family. Studies have shown that depression is mainly associated with presynaptic membrane receptor hypersensitivity and postsynaptic membrane 5-HTIA receptor hypersensitivity. In addition, it can affect the release of other neurotransmitters such as γ-aminobutyric acid (GABA) and DA (47). Dopamine is an important neurotransmitter in the reward system; dopaminergic dysfunction was found to be associated with anhedonia and the development of depression. Dopamine gene polymorphism affects dopamine transmission and receptor binding, leading to increased susceptibility to depression (48). GABA is an inhibitory neurotransmitter expressed by interneurons, the primary function of which is the regulation of local neuronal circuits. The GABA system is particularly important for the self-balance of the brain. Downregulation of GABA is associated with anxiety, depression, tension, insomnia, and other emotional problems. Reduced GABA levels were found in both animal models of depression and patients with depression. GABA antagonists were found to reverse depression-like behaviors, and increased GABA activity could improve depression status (49). The neuroactive ligand-receptor interaction signaling pathway is composed of all receptors and ligands related to intracellular and extracellular signaling pathways in the plasma membrane. Increasing evidence indicates that cyclic guanosine monophosphate (cGMP)-mediated signaling might be a part of neuronal modulation related to depression (50). In addition, studies have shown that cGMP-PKG signaling pathway might have an inhibitory effect on depressive and anxiety-like behaviors related to chronic stress-induced brain oxidative damage and neuronal apoptosis (51).

In this study, we found that stigmasterol, one of the main active compounds of Chaihu, targeted the serotonergic synapse pathways (HTR2A, MAOB, MAOA, PRKACA, and PTGS2), GABAergic synapse pathways (GABRA3 and PRKACA) and dopaminergic synapse pathways (MAOA, MAOB, PRKACA, and SLC6A3). At the same time, beta-sitosterol, one of the main active components of Radix Paeoniae Alba, targeted serotonergic synapse pathways (HTR2A, PTGS2, SLC6A4, PRKACA, PRKCA, and CASP3), GABAergic synapse pathways (GABRA2, GABRA3, GABRA5, PRKACA, and PRKCA) and dopaminergic synapse pathways (DRD1, PRKACA, and PRKCA) in the nervous system. Genetic factors are important internal factors in the development of depression, while inter-individual differences result from genetic diversity. Notably, the genes, targets, and pathways identified in the present study are not only specifically related to depression but also may involve other psychiatric disorders. This is consistent with the largest genetic meta-analysis of research on depression to date, which involved 2.1 million people and identified a possible common genetic component between depression and other psychiatric disorders, including anorexia nervosa, attention deficit hyperactivity disorder, schizophrenia, and bipolar disorder (52). The findings suggested that SNSF might exert its antidepressant effects by regulating the signaling pathway of 5-HT, DA, GABA, and neuroactive ligand receptor interactions, thereby improving neurotransmitter conduction function and neuronal cell growth. The predicted results of the active ingredients and targets in SNSF are in accord with those reported in the literature, which indicates that the active ingredients screening and targets prediction based on network pharmacology are scientific and accurate to a certain extent.

Thus, we used the method of molecular docking to simulate and predict the binding of active components to targets. The results indicated that Molecular docking suggested that beta-sitosterol, nobiletin, 7-methoxy-2-methyl isoflavone bound well to the main potential targets including CREB1, NOS3, CASP3, TP53, ESR1, PTGS2, and SLC6A4. Therefore, these might be the key compounds for the effect against depression. These findings were in accord with the prediction obtained through the network pharmacology approach. Our findings might provide a guidance for further studies on the molecular targets of SNSF in the treatment of depression and the application of network pharmacology in drug development. It may also provide valuable clues for the development of novel TCM antidepressant drugs with good efficacy, low toxicity and multiple targets.

### Limitations

Despite of the strengths, there were still some limitations to the present study. Firstly, studies on the active ingredients determination of the content of SNSF are currently incomplete, therefore, future research on content determination should be carried out. Secondly, the analysis of the main components of the TCM formula and its drug-like properties might not be sufficient enough, and the important targets were identified only on the basis of computer simulation and verification against previous studies involving this type of drugs. Thirdly, whether the role of pathways in our research was up-regulated or down-regulated was unclear. Therefore, further experiments are necessary to explore and verify more pharmacological effects and mechanisms of SNSF.

## Conclusion

In this study, we found that SNSF is a valuable TCM for treating patients with depression through multiple components, targets, and pathways. Its pharmacological mechanism could through the serotoninergic synaptic pathway, dopaminergic synaptic pathway, and GABAergic synapse pathway to alleviate depression. Hopefully, our research may provide a scientific basis for the prodrug discovery of its natural ingredients and the identification of therapeutic targets in the future.

## Data availability statement

The datasets presented in this study can be found in online repositories. The names of the repository/repositories and accession number(s) can be found in the article/Supplementary material.

## Author contributions

XC, QO, and HW contributed to the conception and design of the study. YX, JH, and TX collected the data. XC, JL, and DH performed the statistical analysis. HW wrote the first draft of the manuscript. XC, SW, and SZ critically revised the manuscript. All authors contributed to manuscript revision, read, and approved the final version of the manuscript.

## Footnotes

1
https://tcmsp-e.com/

2
https://www.uniprot.org/

3
https://www.genecards.org/

4
https://www.disgenet.org/

5
http://db.idrblab.net/ttd/

6
https://omim.org/

7
https://go.drugbank.com/

8
https://www.string-db.org/

9
http://geneontology.org/

10
https://www.genome.jp/kegg/

11
http://www.rcsb.org/

12
https://pubchem.ncbi.nlm.nih.gov/
