# Supplementary material for: Potential mechanisms underlying the therapeutic roles of sinisan formula in depression: Based on network pharmacology and molecular docking study
Source: Front Psychiatry. 2022 Nov 9;13:1063489. doi: 10.3389/fpsyt.2022.1063489 (PMC9681910; doi:10.3389/fpsyt.2022.1063489)
Supplement: Supplementary file 1 [file Table_1.DOCX]

**Supplementary Table 1　Active compounds of Sinisan**

| **MOL** ID | **Active compound** | **OB (%**) | **BBB** | **DL** | **Drug** |
| --- | --- | --- | --- | --- | --- |
| MOL001645 | linoleyl acetate | 42.1 | 1.08 | 0.2 | Chaihu(Radix Bupleuri) |
| MOL000449 | stigmasterol | 43.83 | 1 | 0.76 |  |
| MOL004598 | 3,5,6,7-tetramethoxy-2-(3,4,5-trimethoxyphenyl)chromone | 31.97 | 0.08 | 0.59 |  |
| MOL004609 | areapillin | 48.96 | -0.29 | 0.41 |  |
| MOL004624 | longikaurin A | 47.72 | 0.09 | 0.53 |  |
| MOL004653 | (+)-anomalin | 46.06 | 0 | 0.66 |  |
| MOL004718 | α-spinasterol | 42.98 | 0.79 | 0.76 |  |
| MOL000211 | [mairin](https://tcmsp-e.com/molecule.php?qn=211) | 55.38 | 0.22 | 0.78 | Baishao(Paeoniae Radix Alba) |
| MOL000358 | [beta-sitosterol](https://tcmsp-e.com/molecule.php?qn=358) | 36.91 | 0.99 | 0.75 |  |
| MOL000359 | [sitosterol](https://tcmsp-e.com/molecule.php?qn=359) | 36.91 | 0.87 | 0.75 |  |
| MOL013277 | isosinensetin | 51.15 | 0.03 | 0.44 | Zhishi(Aurantii Fructus Immaturus) |
| MOL013279 | 5,7,4'-trimethylapigenin | 39.83 | 0.12 | 0.3 |  |
| MOL013430 | prangenin | 43.6 | 0.29 | 0.29 |  |
| MOL013435 | poncimarin | 63.62 | 0 | 0.35 |  |
| MOL013436 | isoponcimarin | 63.28 | -0.02 | 0.31 |  |
| MOL013437 | 6-methoxy aurapten | 31.24 | 0.38 | 0.3 |  |
| MOL001803 | sinensetin | 50.56 | 0.04 | 0.45 |  |
| MOL001941 | ammidin | 34.55 | 0.92 | 0.22 |  |
| MOL005100 | 5,7-dihydroxy-2-(3-hydroxy-4-methoxyphenyl)chroman-4-one | 47.74 | -0.3 | 0.27 |  |
| MOL005828 | nobiletin | 61.67 | -0.08 | 0.52 |  |
| MOL005849 | didymin | 38.55 | -0.07 | 0.24 |  |
| MOL007879 | Tetramethoxyluteolin | 43.68 | 0.09 | 0.37 |  |
| MOL001484 | [Inermine](https://tcmsp-e.com/molecule.php?qn=1484) | 75.18 | 0.4 | 0.54 | Gancao(licorice) |
| MOL001792 | [DFV](https://tcmsp-e.com/molecule.php?qn=1792) | 32.76 | -0.29 | 0.18 |  |
| MOL000211 | [Mairin](https://tcmsp-e.com/molecule.php?qn=211) | 55.38 | 0.22 | 0.78 |  |
| MOL002311 | [Glycyrol](https://tcmsp-e.com/molecule.php?qn=2311) | 90.78 | -0.2 | 0.67 |  |
| MOL000239 | [Jaranol](https://tcmsp-e.com/molecule.php?qn=239) | 50.83 | -0.22 | 0.29 |  |
| MOL002565 | [Medicarpin](https://tcmsp-e.com/molecule.php?qn=2565) | 49.22 | 0.53 | 0.34 |  |
| MOL000359 | [sitosterol](https://tcmsp-e.com/molecule.php?qn=359) | 36.91 | 0.87 | 0.75 |  |
| MOL003656 | [Lupiwighteone](https://tcmsp-e.com/molecule.php?qn=3656) | 51.64 | -0.23 | 0.37 |  |
| MOL003896 | [7-Methoxy-2-methyl isoflavone](https://tcmsp-e.com/molecule.php?qn=3896) | 42.56 | 0.56 | 0.2 |  |
| MOL000392 | [formononetin](https://tcmsp-e.com/molecule.php?qn=392) | 69.67 | 0.02 | 0.21 |  |
| MOL004805 | [(2S)-2-[4-hydroxy-3-(3-methylbut-2-enyl)phenyl]-8,8-dimethyl-2,3-dihydropyrano[2,3-f]chromen-4-one](https://tcmsp-e.com/molecule.php?qn=4805) | 31.79 | 0.25 | 0.72 |  |
| MOL004806 | [euchrenone](https://tcmsp-e.com/molecule.php?qn=4806) | 30.29 | 0.39 | 0.57 |  |
| MOL004808 | [glyasperin B](https://tcmsp-e.com/molecule.php?qn=4808) | 65.22 | -0.09 | 0.44 |  |
| MOL004810 | [glyasperin F](https://tcmsp-e.com/molecule.php?qn=4810) | 75.84 | -0.15 | 0.54 |  |
| MOL004811 | [Glyasperin C](https://tcmsp-e.com/molecule.php?qn=4811) | 45.56 | 0.07 | 0.4 |  |
| MOL004814 | [Isotrifoliol](https://tcmsp-e.com/molecule.php?qn=4814) | 31.94 | -0.25 | 0.42 |  |
| MOL004815 | [(E)-1-(2,4-dihydroxyphenyl)-3-(2,2-dimethylchromen-6-yl)prop-2-en-1-one](https://tcmsp-e.com/molecule.php?qn=4815) | 39.62 | -0.12 | 0.35 |  |
| MOL004820 | [kanzonols W](https://tcmsp-e.com/molecule.php?qn=4820) | 50.48 | 0.04 | 0.52 |  |
| MOL004828 | [Glepidotin A](https://tcmsp-e.com/molecule.php?qn=4828) | 44.72 | 0.06 | 0.35 |  |
| MOL004829 | [Glepidotin B](https://tcmsp-e.com/molecule.php?qn=4829) | 64.46 | -0.09 | 0.34 |  |
| MOL004833 | [Phaseolinisoflavan](https://tcmsp-e.com/molecule.php?qn=4833) | 32.01 | 0.46 | 0.45 |  |
| MOL004835 | [Glypallichalcone](https://tcmsp-e.com/molecule.php?qn=4835) | 61.6 | 0.23 | 0.19 |  |
| MOL004838 | [8-(6-hydroxy-2-benzofuranyl)-2,2-dimethyl-5-chromenol](https://tcmsp-e.com/molecule.php?qn=4838) | 58.44 | 0.34 | 0.38 |  |
| MOL004848 | [licochalcone G](https://tcmsp-e.com/molecule.php?qn=4848) | 49.25 | -0.04 | 0.32 |  |
| MOL004849 | [3-(2,4-dihydroxyphenyl)-8-(1,1-dimethylprop-2-enyl)-7-hydroxy-5-methoxy-coumarin](https://tcmsp-e.com/molecule.php?qn=4849) | 59.62 | -0.23 | 0.43 |  |
| MOL004855 | [Licoricone](https://tcmsp-e.com/molecule.php?qn=4855) | 63.58 | -0.14 | 0.47 |  |
| MOL004856 | [Gancaonin A](https://tcmsp-e.com/molecule.php?qn=4856) | 51.08 | 0.13 | 0.4 |  |
| MOL004857 | [Gancaonin B](https://tcmsp-e.com/molecule.php?qn=4857) | 48.79 | -0.1 | 0.45 |  |
| MOL004863 | [3-(3,4-dihydroxyphenyl)-5,7-dihydroxy-8-(3-methylbut-2-enyl)chromone](https://tcmsp-e.com/molecule.php?qn=4863) | 66.37 | -0.13 | 0.41 |  |
| MOL004864 | [5,7-dihydroxy-3-(4-methoxyphenyl)-8-(3-methylbut-2-enyl)chromone](https://tcmsp-e.com/molecule.php?qn=4864) | 30.49 | 0.21 | 0.41 |  |
| MOL004866 | [2-(3,4-dihydroxyphenyl)-5,7-dihydroxy-6-(3-methylbut-2-enyl)chromone](https://tcmsp-e.com/molecule.php?qn=4866) | 44.15 | -0.28 | 0.41 |  |
| MOL004879 | [Glycyrin](https://tcmsp-e.com/molecule.php?qn=4879) | 52.61 | -0.13 | 0.47 |  |
| MOL004882 | [Licocoumarone](https://tcmsp-e.com/molecule.php?qn=4882) | 33.21 | 0.06 | 0.36 |  |
| MOL004883 | [Licoisoflavone](https://tcmsp-e.com/molecule.php?qn=4883) | 41.61 | -0.27 | 0.42 |  |
| MOL004884 | [Licoisoflavone B](https://tcmsp-e.com/molecule.php?qn=4884) | 38.93 | -0.18 | 0.55 |  |
| MOL004885 | [licoisoflavanone](https://tcmsp-e.com/molecule.php?qn=4885) | 52.47 | -0.22 | 0.54 |  |
| MOL004891 | [shinpterocarpin](https://tcmsp-e.com/molecule.php?qn=4891) | 80.3 | 0.68 | 0.73 |  |
| MOL004907 | [Glyzaglabrin](https://tcmsp-e.com/molecule.php?qn=4907) | 61.07 | -0.2 | 0.35 |  |
| MOL004908 | [Glabridin](https://tcmsp-e.com/molecule.php?qn=4908) | 53.25 | 0.36 | 0.47 |  |
| MOL004910 | [Glabranin](https://tcmsp-e.com/molecule.php?qn=4910) | 52.9 | 0.31 | 0.31 |  |
| MOL004911 | [Glabrene](https://tcmsp-e.com/molecule.php?qn=4911) | 46.27 | 0.04 | 0.44 |  |
| MOL004912 | [Glabrone](https://tcmsp-e.com/molecule.php?qn=4912) | 52.51 | -0.11 | 0.5 |  |
| MOL004913 | [1,3-dihydroxy-9-methoxy-6-benzofurano[3,2-c]chromenone](https://tcmsp-e.com/molecule.php?qn=4913) | 48.14 | -0.19 | 0.43 |  |
| MOL004915 | [Eurycarpin A](https://tcmsp-e.com/molecule.php?qn=4915) | 43.28 | -0.06 | 0.37 |  |
| MOL004941 | [(2R)-7-hydroxy-2-(4-hydroxyphenyl)chroman-4-one](https://tcmsp-e.com/molecule.php?qn=4941) | 71.12 | -0.25 | 0.18 |  |
| MOL004945 | [(2S)-7-hydroxy-2-(4-hydroxyphenyl)-8-(3-methylbut-2-enyl)chroman-4-one](https://tcmsp-e.com/molecule.php?qn=4945) | 36.57 | -0.04 | 0.32 |  |
| MOL004948 | [Isoglycyrol](https://tcmsp-e.com/molecule.php?qn=4948) | 44.7 | 0.05 | 0.84 |  |
| MOL004957 | [HMO](https://tcmsp-e.com/molecule.php?qn=4957) | 38.37 | 0.25 | 0.21 |  |
| MOL004959 | [1-Methoxyphaseollidin](https://tcmsp-e.com/molecule.php?qn=4959) | 69.98 | 0.48 | 0.64 |  |
| MOL004966 | [3'-Hydroxy-4'-O-Methylglabridin](https://tcmsp-e.com/molecule.php?qn=4966) | 43.71 | 0.73 | 0.57 |  |
| MOL000497 | [licochalcone a](https://tcmsp-e.com/molecule.php?qn=497) | 40.79 | -0.21 | 0.29 |  |
| MOL004974 | [3'-Methoxyglabridin](https://tcmsp-e.com/molecule.php?qn=4974) | 46.16 | 0.47 | 0.57 |  |
| MOL004978 | [2-[(3R)-8,8-dimethyl-3,4-dihydro-2H-pyrano[6,5-f]chromen-3-yl]-5-methoxyphenol](https://tcmsp-e.com/molecule.php?qn=4978) | 36.21 | 0.61 | 0.52 |  |
| MOL004980 | [Inflacoumarin A](https://tcmsp-e.com/molecule.php?qn=4980) | 39.71 | -0.24 | 0.33 |  |
| MOL004985 | [icos-5-enoic acid](https://tcmsp-e.com/molecule.php?qn=4985) | 30.7 | 1.09 | 0.2 |  |
| MOL004988 | [Kanzonol F](https://tcmsp-e.com/molecule.php?qn=4988) | 32.47 | 0.56 | 0.89 |  |
| MOL004989 | [6-prenylated eriodictyol](https://tcmsp-e.com/molecule.php?qn=4989) | 39.22 | -0.29 | 0.41 |  |
| MOL004991 | [7-Acetoxy-2-methylisoflavone](https://tcmsp-e.com/molecule.php?qn=4991) | 38.92 | 0.16 | 0.26 |  |
| MOL004996 | [gadelaidic acid](https://tcmsp-e.com/molecule.php?qn=4996) | 30.7 | 0.94 | 0.2 |  |
| MOL000500 | [Vestitol](https://tcmsp-e.com/molecule.php?qn=500) | 74.66 | 0.3 | 0.21 |  |
| MOL005000 | [Gancaonin G](https://tcmsp-e.com/molecule.php?qn=5000) | 60.44 | 0.23 | 0.39 |  |
| MOL005001 | [Gancaonin H](https://tcmsp-e.com/molecule.php?qn=5001) | 50.1 | -0.14 | 0.78 |  |
| MOL005003 | [Licoagrocarpin](https://tcmsp-e.com/molecule.php?qn=5003) | 58.81 | 0.61 | 0.58 |  |
| MOL005007 | [Glyasperins M](https://tcmsp-e.com/molecule.php?qn=5007) | 72.67 | -0.04 | 0.59 |  |
| MOL005012 | [Licoagroisoflavone](https://tcmsp-e.com/molecule.php?qn=5012) | 57.28 | 0.09 | 0.49 |  |
| MOL005016 | [Odoratin](https://tcmsp-e.com/molecule.php?qn=5016) | 49.95 | -0.24 | 0.3 |  |
| MOL005017 | [Phaseol](https://tcmsp-e.com/molecule.php?qn=5017) | 78.77 | -0.06 | 0.58 |  |
| MOL005018 | [Xambioona](https://tcmsp-e.com/molecule.php?qn=5018) | 54.85 | 0.52 | 0.87 |  |
| MOL005020 | [dehydroglyasperins C](https://tcmsp-e.com/molecule.php?qn=5020) | 53.82 | -0.12 | 0.37 |  |
